# Evaluation of Factors Associated With Health-Related Quality of Life in Patients With Chronic Diseases in Jeddah, Saudi Arabia: A Cross-Sectional Study

**DOI:** 10.7759/cureus.105004

**Published:** 2026-03-10

**Authors:** Raghad O Alsaedi, Mohammed K Al-Hanawi

**Affiliations:** 1 Department of Health Services and Hospital Administration, Faculty of Economics and Administration, King Abdulaziz University, Jeddah, SAU; 2 Department of Pharmaceutical Care, Ministry of National Guard Health Affairs (MNGHA), Jeddah, SAU

**Keywords:** chronic diseases, eq-5d-5l, health-related quality of life (hrqol), multimorbidity, socioeconomic factors

## Abstract

Background

Chronic diseases are emerging as a growing concern in Saudi Arabia, contributing to increased morbidity, mortality, healthcare costs, and reduced health-related quality of life (HRQoL). HRQoL is influenced by disease and treatment and is commonly assessed using the EuroQol five-dimensions five-levels (EQ-5D-5L) instrument. Assessing HRQoL helps in identifying areas that can be improved, ultimately leading to enhanced patient care and outcomes.

Objective

The study aims to evaluate the HRQoL among patients with chronic diseases and to assess the association of sociodemographic, clinical, lifestyle, and disease-related factors with HRQoL in Jeddah, Saudi Arabia.

Methods

A cross-sectional, multicenter study was conducted. Data were obtained using either interview-administered or self-administered questionnaires. The first section included patients’ specific variables, while the second section consisted of the EQ-5D instrument.

Results

The study included 503 participants. The mean age of the participants was 44.3±13.8 years. The mean EQ-5D index score was 0.84±0.23, and the mean EQ-visual analogue scale (EQ-VAS) score was 77.22±20.60, indicating a generally good level of HRQoL at the population level. A strong relationship was observed between increasing chronic disease burden (multimorbidity) and poorer HRQoL across the overall sample, whereas the mean disease duration was not significantly related to HRQoL.

Conclusion

Patients with chronic diseases in Jeddah reported moderate to good HRQoL, the overall mean EQ-5D index score was 0.84±0.23, and the mean EQ-VAS score was 77.22±20.60. More than half of the participants experienced impaired health, especially in pain and psychological well-being. Multimorbidity was the most significant cause of impaired HRQoL, whereas the disease duration was not significantly related to HRQoL.

## Introduction

Chronic diseases are a growing concern, significantly impacting public health and the healthcare system in Saudi Arabia. Conditions such as diabetes, hypertension, cardiovascular diseases, and obesity are prevalent and contribute to high rates of morbidity and mortality and lower quality of life. Chronic diseases affect 37.2% of the global population and 23.3% of the population in Saudi Arabia [1]. The increasing prevalence of chronic diseases causes increasing healthcare expenses, which frequently require costly long-term treatments and present a significant economic challenge to Saudi Arabia's healthcare system [2].

Chronic diseases continue to represent a substantial burden on public health globally, increasing morbidity and mortality worldwide [3]. Improving healthcare services will not only decrease mortality and morbidity but also improve health-related quality of life (HRQoL) for patients with chronic diseases [4].

HRQoL is a self-perceived measure of well-being, which is influenced by the presence of disease or treatment [5]. Instruments used to measure HRQoL are similar in concept to questionnaires. One of the most widely used tools to assess HRQoL is the EuroQol five-dimensions five-levels (EQ-5D-5L) instrument, developed by the EuroQol Group in 1987. Since then, studies have been using this instrument to provide evidence related to HRQoL [6]. The EQ-5D was intended to be a short, simple questionnaire that minimized the burden required for data collection and is suitable for use in various healthcare contexts. EQ-5D assesses health in a way that enables comparisons across different patient groups, diseases, and treatments [5,7].

HRQoL has a major effect on the cost-effectiveness analysis of healthcare strategies. So it has been widely used by researchers in Saudi Arabia [8]. EuroQol five-dimensions five-levels (EQ-5D-5L) health states are commonly converted into utility index scores using a country- or region-specific value set derived from a nationally representative valuation study, which can further be used in cost-effectiveness analyses, providing evidence on resource allocation and healthcare resource funding decisions [9].

Several studies have been conducted to explore and assess the quality of life for patients with chronic diseases. All these studies aimed to generate useful information to guide improved health care policies and interventions [4]. Despite the fact that a large number of studies have evaluated HRQoL among particular patient groups, especially diabetes, in Saudi Arabia, the majority of these studies have been restricted to a single disease, small sample sizes, and single-center designs. Because of this, there is insufficient evidence describing HRQoL among patients with chronic diseases in Jeddah, especially when considering the combined burden of multiple chronic conditions.

This study aims to evaluate the HRQoL among patients with chronic diseases and to assess the association of sociodemographic, clinical, lifestyle, and disease-related factors with HRQoL in Jeddah, Saudi Arabia.

By focusing on HRQoL, these studies help in identifying areas that can be improved, ultimately leading to enhanced patient care and outcomes. This increasing amount of research emphasizes the value of HRQoL assessments in managing chronic diseases and the necessity of ongoing initiatives to improve the health and well-being of affected individuals in Saudi Arabia.

## Materials and methods

Study design and setting

This study adopts a cross-sectional design to evaluate HRQoL, its associated factors, and examine associations between HRQoL and selected sociodemographic, clinical, and lifestyle factors among patients with chronic diseases in Jeddah, Saudi Arabia.

A stratified geographic sampling approach was applied by dividing Jeddah into five regions (North, South, East, West, and Central). Within each region, two primary healthcare centers with the highest patient flow were selected, yielding 10 healthcare centers in total. This strategy is designed to optimize the recruitment efficiency and to allow a wide range of patient populations to be included in this study, thereby strengthening the generalizability of the findings.

The minimum sample size for this cross-sectional study was calculated using the standard formula: $n = \frac{Z^{2} \times p \times (1 - p)}{d^{2}}$. The estimated minimum sample size was 385 participants, assuming a 95% confidence level, a 5% margin of error, and the maximum variability (p=0.5).

Study population

The study included patients with chronic diseases, able to understand and complete the questionnaire, aged 18 years or older, of both genders having at least one of the common chronic diseases in Saudi Arabia, who had been diagnosed at least in the last six months, and who were attending selected primary healthcare centers during the study period. The participants were excluded if they were younger than 18 years, recently diagnosed (within less than six months), were pregnant women, or refused to participate.

Data collection procedure

**Figure 1:**
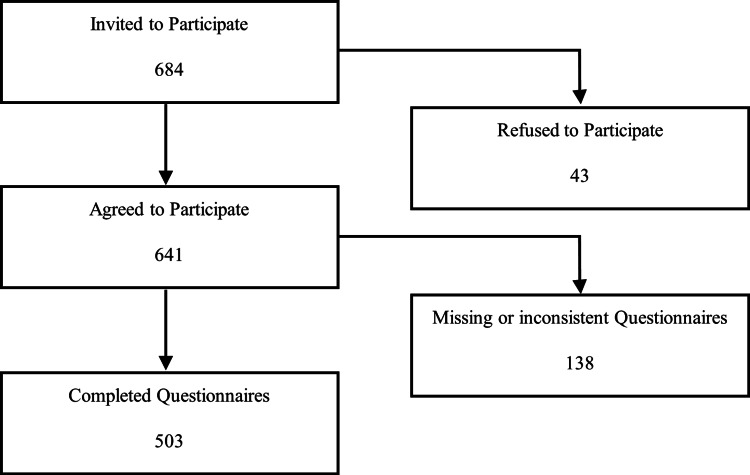
Flow Diagram of Study Participants

Data was obtained using an interview-administered or self-administered questionnaire. The data collection was conducted over a one-month period in November 2025. Prior to data collection, data collectors received standardized training to ensure accurate data collection and proper handling of study procedures. Data were collected through visits to selected primary healthcare centers in Jeddah, Saudi Arabia. Patients attending the selected healthcare centers during the study period were invited to participate, and informed consent was obtained from all participants. Completed questionnaires were reviewed for completeness, and responses with substantial missing or inconsistent data were excluded from the final analysis (Figure 1).

Data collection tools

Data were collected using a structured questionnaire adapted from a previously published study [4]. The original questionnaire consisted of two major sections. The first section is sociodemographic, lifestyle, and clinical information. The second section was designed to measure HRQoL, using Arabic and English versions of EQ-5D-5L. Permission to use the questionnaire was obtained from the original author. Minor modifications were made without altering the core content of the questionnaire.

EQ-5D-5L instrument

Assessing HRQoL was done by using Arabic and English versions of EQ-5D-5L created by the EuroQoL group. The questionnaire was used under license from the EuroQoL Research Foundation. EQ-5D assesses health in a way that enables comparisons across different patient groups, diseases, and treatments [5]. The EQ-5D was designed to be a brief questionnaire that reduces the burden of data collection and is suitable for use in various healthcare contexts [6]. The "5D" in its name reflects the five dimensions it uses to describe health states: mobility, usual activities, self-care, pain and discomfort, and anxiety and depression. Each dimension consists of five levels (1, 2, 3, 4, and 5) indicating no problem, slight problems, moderate problems, severe problems, unable to/extreme problems, respectively [7]. The participants were asked to indicate their health state by selecting the most suitable response level for each of the five dimensions. Each response was recorded as a single-digit number representing the chosen severity level for that dimension. EQ-5D consists of two parts: the first part is the questionnaire, and the second part is the visual analogue scale (EQ-VAS) [7]. In EQ-VAS, the participants were asked to label their health on a scale from 0 to 100, where 100 indicates “The best health you can imagine” and 0 indicates “The worst health you can imagine” on the day of the interview [7].

EQ-5D-5L health states were converted into preference-based utility index scores using the Saudi-specific EQ-5D-5L value set derived from a nationally representative valuation study employing a hybrid heteroskedastic model without a constant [10]. Utility scores were calculated by assigning a value of 1.00 to full health (health state 11111) and subtracting the sum of dimensions and level-specific disutility coefficients corresponding to deviations from level 1 across the five EQ-5D-5L dimensions. This scoring approach yields index values anchored at 1.00 for full health, with negative values indicating health states considered worse than death. In this study, EQ-5D index scores were treated as continuous variables and served as the primary outcome measure of HRQoL, with higher scores reflecting better HRQoL [10]. In addition to the EQ-5D index score, participants evaluated their overall health using the EQ-VAS. The EQ-VAS is a vertical, self-rated scale ranging from 0, representing the worst imaginable health state, to 100, representing the best imaginable health state [7]. EQ-VAS scores were analyzed as a secondary outcome of HRQoL.

Data analysis

Statistical analyses were conducted using IBM SPSS Statistics for Windows, Version 26 (Released 2018; IBM Corp., Armonk, New York, United States), with Microsoft Excel (Microsoft Corp., Redmond, WA, USA) used for data management and preliminary tabulation. Descriptive statistics were used to summarize study variables, with continuous variables presented as mean±standard deviation and categorical variables expressed as frequencies and percentages. Inferential analyses were performed to examine associations between HRQoL outcomes and study variables. Independent samples t-tests were used to compare HRQoL scores between two groups, and one-way analysis of variance (ANOVA) was applied for comparisons involving more than two groups. Chi-square test was used to examine the association between each chronic disease and the number of comorbid conditions (<2 versus ≥2 comorbidities). Spearman's rank correlation analysis was used to assess relationships between continuous or ordinal variables.

To identify independent predictors of HRQoL, stepwise multiple linear regression analysis was performed with the EQ-5D index score entered as the dependent variable. Sociodemographic, clinical, lifestyle, and disease-related variables were included as candidate predictors. Model assumptions were evaluated through collinearity diagnostics, including tolerance and variance inflation factor (VIF) values. Regression coefficients (B), standardized coefficients (β), t-statistics, and corresponding p-values were reported. Statistical significance was set at p<0.05.

Ethical considerations

Prior to data collection, approval was obtained from the Institutional Review Board (IRB) Committee of the Ministry of Health, Jeddah (approval no: A02260 dated February 11, 2026). At the beginning of the survey, all participants were given a short explanation of what we intended to explore and informed about how data would be treated with anonymity and confidentiality. Informed, written consent was obtained from each participant who agreed to participate in this study before administering the questionnaire.

## Results

Table 1 summarizes the sociodemographic characteristics of the 503 participants included in the study. The mean age of the study sample was 44.3±13.8 years, indicating a predominantly middle-aged group, which is consistent with the age group most commonly affected by chronic diseases. In terms of gender distribution, men represent the majority of participants (300, 59.6%). With regard to nationality, the majority of participants were Saudi (423, 84.1%). This distribution reflects the demographic composition of the population in Jeddah and supports that our findings are relevant to the local Saudi healthcare status. As for their marital status, most participants were married (364, 72.4%), followed by those who were single (87, 17.3%). Regarding educational level, nearly half of the participants had a high school education or below (248, 49.8%), while a comparable percentage held a bachelor’s degree (240, 48.2%), reflecting the limited representation of advanced education among study participants. Nearly half of the participants were employed (255, 50.9%), more than one-fourth were unemployed (137, 27.3%), and less than 100 were retired (85, 17.0%). Then, 24 (4.8%) were students. This variation reflects diverse occupational backgrounds and economic stability, which may affect access to healthcare and overall well-being. The findings revealed that the distribution of participants by income showed a large percentage of low-income groups, with 153 (46.9%) earning less than 5,000 SAR per month.

**Table 1:** Sociodemographic Characteristics of Participants SD: standard deviation; SAR: Saudi Riyal

Item	n	Percent (%)
Total Sample	503	
Age, Mean±SD, (years): 44.3±13.8		
<30 Years (Adult)	93	18.49
30-60 years (Middle age)	325	64.61
>60 years (Elderly)	84	16.70
Gender		
Male	300	59.60
Female	203	40.40
Nationality		
Saudi	423	84.10
Non-Saudi	80	15.90
Marital Status		
Single	87	17.30
Married	364	72.40
Divorced	32	6.40
Widowed	20	4.00
Education Level		
High school or below	248	49.80
Bachelor's degree	240	48.20
Postgraduate degree	10	2.00
Employment Status		
Employed	255	50.90
Unemployed	137	27.30
Retired	85	17.00
Student	24	4.80
Monthly Income (SAR)		
<5,000 SAR	153	46.90
5,000-9,999 SAR	99	30.40
10,000-14,999 SAR	41	12.60
≥15,000 SAR	33	10.10

Table 2 summarizes the anthropometric, lifestyle, and clinical characteristics of the participants, and shows a population with a high prevalence of chronic diseases. The mean BMI was 28.1±5.8 kg/m^2^, indicating that most patients were overweight. According to the WHO BMI classification, most participants were either overweight (194, 38.6%) or obese (160, 31.8%). Regarding lifestyle characteristics, the majority of participants were non-smokers (353, 70.2%), while 150 (29.8%) were current smokers, with a mean smoking duration of 16.0±10.3 years. The most reported smoking method was cigarettes (107, 71.3%).

**Table 2:** Anthropometric, Lifestyle, and Clinical Characteristics of Participants ^# ^Calculated among participants with the condition only. BMI: body mass index

Items	n	Percent (%)
Anthropometric Characteristics: BMI Mean±SD (kg/m^2^): 28.1±5.8		
BMI WHO Classification		
Underweight	9	1.8
Normal Weight	128	25.4
Overweight	194	38.6
Obesity	160	31.8
Lifestyle Characteristics		
Smoking Status		
Non-smoker	353	70.2
Smoker	150	29.8
Smoking Duration (years), Mean±SD^#^: 16.0±10.3		
Smoking Method (among smokers)		
Cigarette	107	71.3
Shisha	23	15.3
E-cigarette/Vape	7	4.7
Mixed methods	13	8.7
Clinical Characteristics		
Diabetes Mellitus		
Type 1 diabetes	105	20.9
Type 2 diabetes	153	30.4
Hypertension	224	44.5
Cardiovascular Conditions		
Heart failure	21	4.2
Hyperlipidemia	66	13.1
Heart diseases	39	7.8
Cardiovascular disease	9	1.8
Arteriosclerosis	8	1.6
Venous thromboembolism	7	1.4
Gastrointestinal Conditions		
Irritable bowel syndrome	74	14.7
Crohn’s disease	7	1.4
Celiac disease	3	0.6
Other Chronic Conditions		
Asthma	60	11.9
Migraine	30	6.0
Rheumatoid arthritis	27	5.4
Renal failure	8	1.6
Viral hepatitis	3	0.6
Multiple sclerosis	1	0.2
Other chronic disease	34	6.8
Cancer Diagnosis	4	0.8
Years with cancer, Mean±SD^#^:7.7±9.9		
Disability	8	1.6
Health Insurance		
No	376	75.0
Yes	125	25.0

Clinically, diabetes mellitus was the highest prevalent disease among the study participants, with 105 (20.9%) participants reporting type 1 diabetes and 153 (30.4%) type 2 diabetes, followed by hypertension (224, 44.5%). Only a small proportion of participants reported having cancer or disability; for those with cancer, the mean disease duration was 7.7±9.9 years. Finally, the majority of participants (376, 75.0%) had no health insurance, suggesting potential barriers to healthcare access.

Table 3 presents the self-reported duration of major chronic diseases among participants. Overall, the findings indicate that participants had been experiencing chronic conditions for extended periods, representing a long-term disease burden for them. Diabetes mellitus showed a mean disease duration of 10.57±8.49 years, with a comparable duration between type 1 diabetes (9.84±9.47 years) and type 2 diabetes (11.13±7.51 years), indicating prolonged disease exposure. Hypertension and hyperlipidemia were also long-standing conditions with a mean duration of 9.63±6.66 years and 8.35±5.53 years, respectively. Asthma demonstrated the longest mean disease duration (21.30±10.79 years) among all the diseases due to its common occurrence in childhood.

**Table 3:** Disease Duration Among Participants With Chronic Diseases Note: Disease duration refers to the self-reported duration of the specific chronic disease and was calculated only among participants who reported duration data.

Chronic Disease	n	Disease Duration (Years), Mean±SD
Diabetes Mellitus (DM)	206	10.57±8.49
DM Type 1	89	9.84±9.47
DM Type 2	117	11.13±7.51
Hypertension	157	9.63±6.66
Hyperlipidemia	46	8.35±5.53
Irritable Bowel Syndrome	53	8.75±5.34
Asthma	44	21.30±10.79
Rheumatoid Arthritis	15	9.13±5.51
Heart Diseases	32	6.69±5.44

Table 4 presents HRQoL using the EQ-5D-5L instrument across its five dimensions. Overall, the findings indicate a generally good health status, with most participants reporting no problems in all dimensions. In the mobility dimension, the majority of participants (409, 81.3%) reported no mobility problems, but not a single participant was unable to walk. Self-care was the least affected dimension, with 446 (88.7%) participants reporting no problems at all, with only three (0.6%) reporting unable to wash or dress. Regarding usual activities, approximately three-quarters of participants (385, 76.5%) reported no problems, and five (1.0%) were unable to perform usual activities. On the other hand, pain/discomfort emerged as one of the most affected dimensions. While 259 (51.5%) participants reported no pain, nearly half experienced some level of pain or discomfort, including 83 (16.5%) with slight pain, 104 (20.7%) with moderate pain, 40 (8.0%) with severe pain, and 17 (3.4%) with extreme pain. Similarly, in the anxiety/depression dimension, 183 (36.4%) participants experienced some degree of psychological distress.

**Table 4:** EQ-5D-5L Responses EQ-5D-5L: EuroQol five-dimensions five-levels (EQ-5D-5L) instrument

EQ-5D Dimension	n	Percent (%)
Mobility		
No problems	409	81.3
Slight problems	68	13.5
Moderate problems	20	4.0
Severe problems	6	1.2
Unable to walk	0	0
Self-care		
No problems	446	88.7
Slight problems	22	4.4
Moderate problems	17	3.4
Severe problems	15	3.0
Unable to wash or dress	3	0.6
Usual Activities		
No problems	385	76.5
Slight problems	70	13.9
Moderate problems	26	5.2
Severe problems	17	3.4
Unable to do usual activities	5	1.0
Pain/Discomfort		
No pain	259	51.5
Slight pain	83	16.5
Moderate pain	104	20.7
Severe pain	40	8.0
Extreme pain	17	3.4
Anxiety/Depression		
Not anxious/depressed	320	63.6
Slightly anxious/depressed	80	15.9
Moderately anxious/depressed	74	14.7
Severely anxious/depressed	21	4.2
Extremely anxious/depressed	8	1.6

Table 5 presents a comprehensive overview of HRQoL outcomes among the study participants based on the EQ-5D-5L descriptive system. Following the assessment, 301 (59.8%) participants were classified as having imperfect health, and 193 (38.4%) had perfect health. Only a few participants (9, 1.8%) reported health states worse than dead (negative value), indicating very poor levels of health in a small proportion of the population. No participants were classified in the death health state (equal to zero).

**Table 5:** Most Reported Health State and Its Frequency by EQ-5D-5L Notes: EQ-5D index score: 0 (death), less than 1 (imperfect health), equal to 1 (perfect/full health), and negative values (worse than dead). EQ-5D-5L: EuroQol five-dimensions five-levels (EQ-5D-5L) instrument

Categories of Health State	n	Percent (%)
Perfect health	193	38.40
Imperfect health	301	59.80
Death	0	0
Worse than dead	9	1.80
Mean Score of 5-Dimensions	Mean±SD	
Mobility score	1.24±0.60	
Self-care score	1.19±0.75	
Usual activity score	1.37±0.80	
Pain/discomfort score	2.05±1.22	
Anxiety/depression score	1.66±1.07	
The Most Frequent Health State	n	%
11111	193	38.40
11121	26	5.20
11131	22	4.40
11112	19	3.80
11122	18	3.60
11113	14	2.80
11133	12	2.40
11132	11	2.20
11211	9	1.80
11221	9	1.80
21231	6	1.20
The Worst Health State Reported in the Study	n	%
45544	1	0.20
45143	1	0.20
44541	1	0.20
44444	1	0.20
44354	1	0.20

The five dimensions of the EQ-5D analysis demonstrated that pain/discomfort was significantly the most affected dimension, with the highest mean score (2.05±1.22), indicating a substantial burden of physical symptoms. This was followed by anxiety/depression (1.66±1.07) and usual activities (1.37±0.80), indicating significant psychological and functional impairments. In contrast, self-care (1.19±0.75) and mobility (1.24±0.60) were the least affected dimensions, indicating largely preserved functional independence in these areas.

Regarding health state profiles, the most frequently reported state was 11111 by 193 (38.4%) participants, representing no problems across all five dimensions. Other commonly reported states reflected mild impairments, particularly in pain/discomfort and anxiety/depression (e.g., 11121, 11131, 11112, and 11122). In contrast, the most severe health states (e.g., 45544, 45143, 44541, 44444, and 44354) were extremely rare, each reported by only one participant (0.2%).

**Figure 2:**
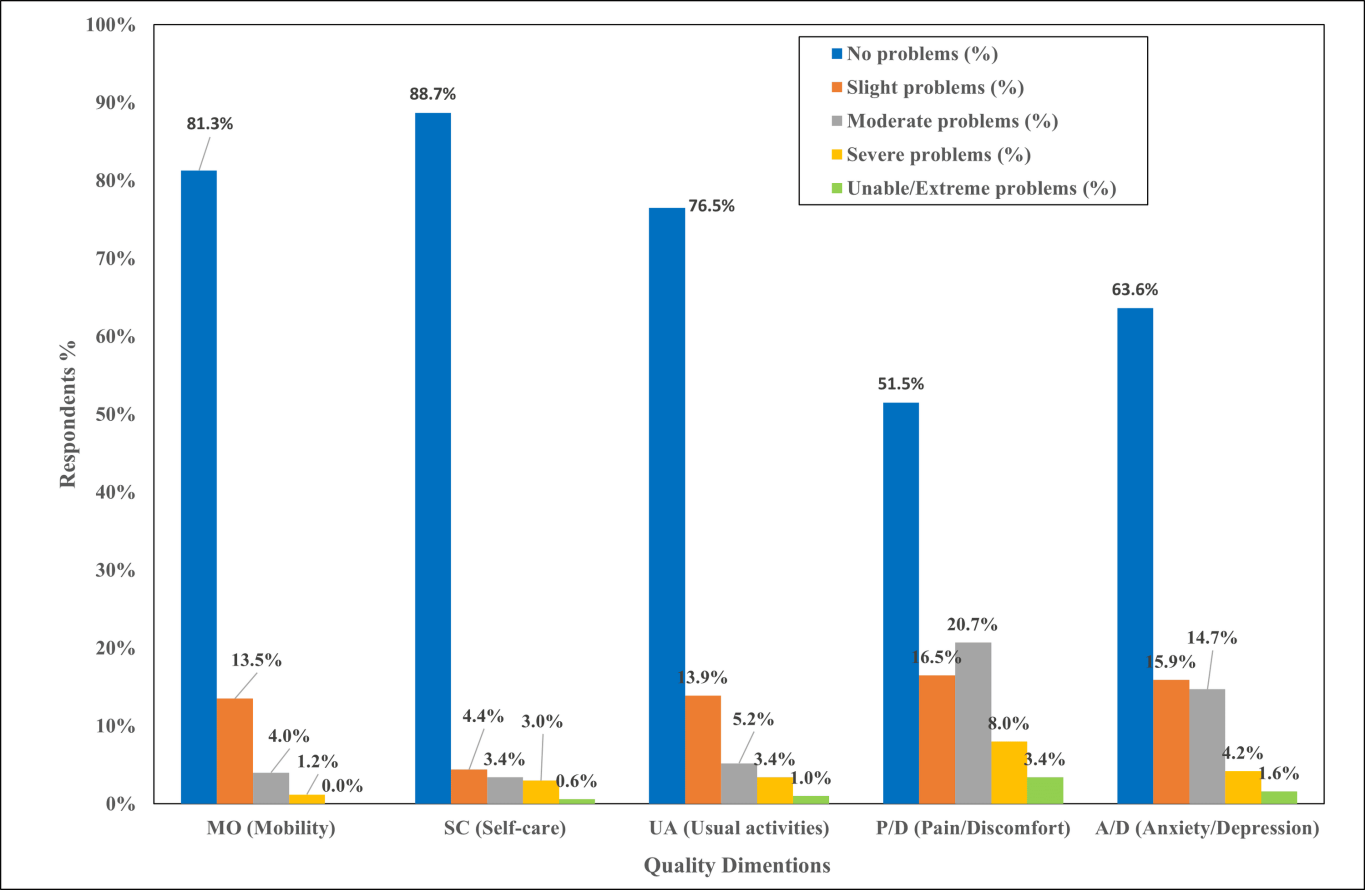
Distribution of EQ-5D-5L Responses Across Health Dimensions EQ-5D-5L: EuroQol five-dimensions five-levels (EQ-5D-5L) instrument

Figure 2 provides a visual summary of the distribution of EQ-5D-5L responses across the five HRQoL dimensions, clearly illustrating patterns of perceived health status within the study population. Mobility and self-care showed the most favorable profiles, with a majority of participants having no problem (409, 81.3%) in the mobility dimension, 447 (88.7%) in the self-care dimension, and only small percentages experiencing severe or extreme limitations, suggesting that physical independence was largely preserved among the participants. Usual activities also demonstrate relatively good results, although a greater proportion of participants reported slight to moderate problems compared with mobility and self-care. In contrast, pain/discomfort was the most affected dimension, with only about half of the participants reporting no problems, and a substantial proportion experiencing moderate to severe or extreme pain, supporting that pain is an important factor contributing to decreased HRQoL. Anxiety/depression similarly shows a notable burden, with roughly one-third of participants reporting some level of psychological distress, ranging from slight to extreme, reflecting the importance of mental health concerns in this population. Overall, the figure demonstrates that impairments in HRQoL were primarily driven by pain and psychological symptoms rather than limitations in basic physical functioning.

Table 6 presents the association between sociodemographic characteristics and HRQoL as measured by the EQ-5D index and EQ-VAS among the study participants. Overall, the mean EQ-5D index score of 0.84±0.23 and EQ-VAS score of 77.22±20.60 indicate a generally good perceived health.

**Table 6:** Association of Sociodemographic Characteristics of the Study Participants With HRQoL p-values shown only once per variable, based on the correct test: Independent samples t-test for binary variables (Gender, Nationality, Health Insurance). One-way analysis of variance (ANOVA) for variables with >2 categories (Age group, Marital Status, Employment Status, Monthly Income, BMI category). *Statistically significant at p<0.05. Denominators differ by variable due to missing data (e.g., Age has 1 missing; Income has many missing). HRQoL: health-related quality of life; BMI: body mass index

Variable	n	EQ-5D Index (Mean±SD)	p-value (EQ-5D)	EQ-VAS Score (Mean±SD)	p-value (EQ-VAS)
Overall HRQoL Score	503	0.84±0.23		77.22±20.60	
Gender	503		<0.001*		0.198
Male	300	0.87±0.20		78.19±19.43	
Female	203	0.80±0.25		75.78±22.19	
Age (years)	502		<0.001*		<0.001*
<30 Years (Adult)	93	0.89±0.16		87.17±14.37	
30-60 years (Middle age)	325	0.85±0.22		75.92±20.65	
>60 years (Elderly)	84	0.75±0.30		71.07±22.71	
Nationality	503		0.030*		0.721
Saudi	423	0.83±0.24		77.08±21.20	
Non-Saudi	80	0.89±0.14		77.97±17.19	
Marital Status	503		<0.001*		<0.001*
Single	87	0.88±0.18		86.52±13.98	
Married	364	0.85±0.23		77.21±20.03	
Widowed	20	0.70±0.25		66.00±20.97	
Divorced	32	0.78±0.26		68.44±25.08	
Employment Status	501		0.001*		<0.001*
Employed	255	0.88±0.16		78.67±19.16	
Unemployed	137	0.80±0.27		74.70±22.01	
Retired	85	0.78±0.29		73.38±22.23	
Student	24	0.86±0.19		90.04±15.11	
Monthly Income (SAR)	326		0.027*		0.454
<5,000	153	0.78±0.28		72.99±22.95	
5,000-9,999	99	0.82±0.23		74.13±22.75	
10,000-14,999	41	0.91±0.13		80.50±18.07	
15,000-19,999	17	0.89±0.18		79.29±19.52	
20,000-29,999	13	0.82±0.23		75.69±24.32	
≥30,000	3	0.62±0.17		68.33±10.41	
Health Insurance	501		0.566		0.931
No	376	0.84±0.23		77.19±20.98	
Yes	125	0.83±0.23		77.00±19.50	

Gender was significantly associated with the EQ-5D index, with males reporting higher HRQoL than females (p < 0.001), although no significant difference was observed in EQ-VAS scores. Age showed a strong association with both HRQoL measures, as younger participants (<30 years) reported the highest scores, while older participants (>60 years) reported the lowest (p<0.001). Nationality was significantly associated with the EQ-5D index, with non-Saudi participants reporting slightly higher EQ-5D index scores, though EQ-VAS scores did not differ significantly.

Marital status was significantly associated with both HRQoL measures, with single participants reporting higher scores than widowed or divorced participants. Employment status also showed a significant association, with employed participants and students reporting higher scores, while unemployed and retired participants had lower scores. Monthly income was significantly associated with the EQ-5D index, showing that the higher monthly income group had better HRQoL; however, no significant association was observed with EQ-VAS scores. It is important to note that not all participants reported their income. Income data was available for only 326 out of 503 participants. Therefore, the analysis examining the association between income and HRQoL was conducted on a reduced sample size. Health insurance status was not significantly associated with either HRQoL measures.

Table 7 presents the association between lifestyle characteristics and HRQoL as measured by the EQ-5D index and EQ-VAS scores within the study participants. Participants with underweight and normal weight reported the highest EQ-5D index and EQ-VAS scores, whereas overweight and obese participants reported progressively lower scores, indicating worsening functional health status and self-perceived health with increasing body weight. Although the association between BMI and the EQ-5D index did not reach statistical significance (p=0.063), BMI was significantly associated with EQ-VAS scores (p<0.001), suggesting that excess body weight has a stronger influence on subjective health perception than on index-based HRQoL measures.

**Table 7:** Association of Lifestyle Characteristics With HRQoL * p-values were derived using one-way ANOVA for BMI categories and independent samples t-tests for smoking status comparisons. HRQoL: health-related quality of life; ANOVA: analysis of variance; EQ-5D: EuroQol five-dimensions; VAS: visual analogue scale

Lifestyle Characteristic	Category	n	EQ-5D Index (Mean±SD)	p-value*	EQ-VAS Score (Mean±SD)	p-value*
Body Mass Index (WHO)	Underweight	9	0.91±0.20	0.063	91.1±8.6	<0.001
	Normal weight	128	0.87±0.20		81.9±18.9	
	Overweight	194	0.86±0.22		77.8±20.4	
	Obese	160	0.81±0.24		71.9±21.5	
Smoking Status	Non-smoker	353	0.84±0.24	0.979	78.5±19.9	0.046
	Smoker	150	0.84±0.20		74.3±22.0	

The EQ-5D index scores of smokers and non-smokers did not differ significantly (p=0.979), suggesting that both groups had comparable functional health status. However, compared to smokers, non-smokers reported significantly higher EQ-VAS scores (p=0.046), indicating poorer self-rated health among smoker participants.

Table 8 presents the association between major chronic diseases and HRQoL as measured by the EQ-5D index. Overall, diabetes mellitus affected more than half of the participants (258, 51.3%) and was associated with a relatively high mean EQ-5D index score of 0.85±0.22. When comparing participants with and without overall diabetes, no significant difference was observed in EQ-5D index scores. However, stratification by diabetes type revealed significant differences: participants with type 1 diabetes reported higher EQ-5D scores (0.92±0.16, p<0.001), whereas those with type 2 diabetes had significantly lower EQ-5D scores (0.80±0.25, p=0.004).

**Table 8:** Major Chronic Diseases and HRQoL * p-values derived from independent-samples t-tests comparing participants with and without the specified chronic disease. HRQoL: health-related quality of life; EQ-5D: EuroQol five-dimensions

Chronic Disease	n (%)	EQ-5D Index (Mean±SD)	p-value*
Diabetes Mellitus (DM)	258 (51.3)	0.85±0.22	0.52
DM Type 1	105 (20.9)	0.92±0.16	<0.001
DM Type 2	153 (30.4)	0.80±0.25	0.004
Hypertension	224 (44.5)	0.80±0.26	<0.001
Hyperlipidemia	66 (13.1)	0.78±0.25	0.012
Irritable Bowel Syndrome	74 (14.7)	0.82±0.23	0.464
Asthma	60 (11.9)	0.85±0.22	0.748
Rheumatoid Arthritis	27 (5.4)	0.62±0.36	<0.001
Heart Diseases	39 (7.8)	0.70±0.30	<0.001

Participants with hypertension and hyperlipidemia demonstrated significantly reduced EQ-5D index scores (0.80±0.26 and 0.78±0.25, respectively), highlighting the negative effect of these conditions on HRQoL. In contrast, conditions such as irritable bowel syndrome and asthma were not significantly associated with lower HRQoL. Participants with rheumatoid arthritis showed an EQ-5D index score of 0.62 ± 0.36, while participants with heart diseases had a score of 0.70 ± 0.30, reflecting the negative effect of these conditions on HRQoL.

Table 9 presents the distribution of participants with selected chronic diseases according to the number of comorbid conditions (<2 versus ≥2). Overall, the majority of participants were classified as having two or more comorbid conditions, indicating a high prevalence of multimorbidity. For diabetes mellitus, the participants are significantly higher in ≥2 comorbid conditions group (179, 72.5%), suggesting a strong association between diabetes and higher comorbidity burden (p<0.001) when stratified by diabetes type, no significant difference was observed for type 1 diabetes (p=0.923), whereas type 2 diabetes was significantly more prevalent among participants with ≥2 comorbid conditions (127, 51.4% versus 26, 10.2%, p<0.001). There was a significant relationship between hypertension and the number of comorbidities, with more prevalent cases in participants with ≥2 comorbid conditions (170, 68.8%) compared with those with fewer than two (54, 21.1%, p<0.001). Hyperlipidemia, irritable bowel syndrome, rheumatoid arthritis, and heart diseases were also significantly higher among participants with ≥2 comorbid conditions (p<0.05 for all), indicating the prevalence of these conditions within individuals with multimorbidity.

**Table 9:** Distribution of Participants by Number of Comorbid Conditions Across Major Chronic Diseases Data are presented as numbers (percentages). * p-values were calculated using the chi-square test to examine the association between each chronic disease and the number of comorbid conditions.

Chronic Disease	<2 Comorbid Conditions	≥2 Comorbid Conditions	Total	p-value
Diabetes Mellitus (DM)	79 (30.9%)	179 (72.5%)	258	<0.001*
DM Type 1	53 (20.7%)	52 (21.1%)	105	0.923
DM Type 2	26 (10.2%)	127 (51.4%)	153	<0.001*
Hypertension	54 (21.1%)	170 (68.8%)	224	<0.001*
Hyperlipidemia	6 (2.3%)	60 (24.3%)	66	<0.001*
Irritable Bowel Syndrome	16 (6.3%)	58 (23.5%)	74	<0.001*
Asthma	38 (14.8%)	22 (8.9%)	60	0.040*
Rheumatoid Arthritis	7 (2.7%)	20 (8.1%)	27	0.008*
Heart Diseases	7 (2.7%)	32 (13.0%)	39	<0.001*

In contrast, asthma demonstrated a different pattern with a higher proportion of cases reported among participants with fewer than two comorbid conditions (38, 14.8%) compared with ≥2 conditions (22, 8.9%), although this difference remains statistically significant (p=0.040).

Table 10 demonstrates a strong inverse association of chronic disease burdens with HRQoL, which reflects considerable overall impact of multimorbidity on both functional health and self-perceived well-being. The highest EQ-5D index and EQ-VAS scores were found in those presenting with a low disease burden (one chronic condition), who constituted the largest proportion of the sample, suggesting relatively preserved quality of life. HRQoL measures declined as the number of chronic conditions increased.

**Table 10:** HRQoL by Number of Chronic Conditions * p-values were derived using ANOVA. Chronic disease burden represents the total number of self-reported diagnosed chronic conditions per participant. HRQoL: health-related quality of life; ANOVA: analysis of variance; EQ-5D: EuroQol five-dimensions; VAS: visual analogue scale

Chronic Disease Burden	Definition	n (%)	EQ-5D Index (Mean±SD)	EQ-VAS Score (Mean±SD)
Low Burden	1 Chronic Condition	256 (50.9)	0.89±0.20	81.7±19.0
Moderate Burden	2 Chronic Conditions	164 (32.6)	0.86±0.16	78.1±18.1
High Burden	≥3 Chronic Conditions	83 (16.5)	0.71±0.31	63.6±21.2
Overall p-value*	-	-	<0.001	<0.001

Participants with a high disease burden (three or more chronic conditions) showed the most pronounced deterioration and reported markedly lower EQ-5D index and EQ-VAS scores, indicating significant impairment in daily functioning and overall health status. Across all disease burden categories, increasing multimorbidity was linked to lower HRQoL and demonstrated statistically significant differences in both measures (p<0.001).

Table 11 presents the Spearman correlation analysis examining the relationships between mean disease duration, comorbidity burden, and HRQoL, and also the correlation between EQ-5D and EQ-VAS scores. The findings indicate that mean disease duration was not significantly correlated with either the EQ-5D index score (ρ=0.06, p=0.181) or the EQ-VAS score (ρ=0.015, p=0.738), suggesting that the length of time individuals have lived with chronic diseases alone does not substantially influence their current functional health status or self-perceived health.

**Table 11:** Spearman Correlation Analysis Between Disease Duration, Comorbidity Burden, and HRQoL Spearman’s rank correlation coefficient (ρ) was used due to the non-normal distribution of clinical variables. ** Correlation is significant at p < 0.001 (two-tailed). Mean disease duration represents the average duration (years) across all reported chronic conditions per participant. HRQoL: health-related quality of life; EQ-5D: EuroQol five-dimensions; VAS: visual analogue scale

Variable 1	Variable 2	Spearman Correlation Coefficient (ρ)	p-value	Significance
Mean Disease Duration (years)	EQ-5D Index Score	0.06	0.181	Not Significant
	EQ-VAS Score	0.015	0.738	Not Significant
Number of Comorbidities	EQ-5D Index Score	-0.307**	<0.001	Highly Significant
	EQ-VAS Score	-0.308**	<0.001	Highly Significant
Number of Comorbidities	Mean Disease Duration (years)	0.003	0.941	Not Significant
EQ-5D Index Score	EQ-VAS Score	0.581**	<0.001	Highly Significant

In contrast, the number of comorbidities demonstrated a moderate and highly significant negative correlation with both HRQoL measures. As comorbidity burden increased, EQ-5D index scores (ρ=-0.307, p<0.001) and EQ-VAS scores (ρ=-0.308, p<0.001) decreased, indicating a marked decline in both objective and subjective quality of life with increasing multimorbidity. Notably, no significant correlation was observed between comorbidity burden and mean disease duration (ρ=0.003, p=0.941), which also indicates that multimorbidity is not only a consequence of longer disease duration but represents the cumulative burden and complexity contributed by multiple co-morbid chronic diseases.

Consequently, a strong, positive, and highly significant correlation was found between the EQ-5D index score and the EQ-VAS score (ρ=0.581, p<0.001), supporting the internal consistency and convergent validity of these two HRQoL measures in capturing related but complementary dimensions of health status. Overall, these findings highlight that comorbidity burden, rather than disease duration, is more strongly associated with reduced HRQoL.

Table 12 represents a stepwise multivariable linear regression analysis. This was conducted to identify independent factors associated with EQ-5D index scores (n=495). Stepwise regression included all listed sociodemographic, clinical, lifestyle, and disease predictors. Only variables that significantly improved model fit were retained.

**Table 12:** Stepwise Linear Regression Analysis of Factors Associated With EQ-5D Index Scores (n=495) B: unstandardized regression coefficient, indicates the expected change in EuroQol five-dimensions (EQ-5D) index score for a one-unit increase in the predictor, controlling for other variables. SE: standard error of the unstandardized coefficient. Standardized β: standardized regression coefficient, shows the relative contribution of each predictor to the model. t: t-statistic testing whether the coefficient differs significantly from zero. p-value indicates statistical significance; values <0.05 are considered significant. VIF: variance inflation factor; values <5 suggest minimal multicollinearity. Reference categories: Male (Gender), Married (Marital Status), No (Rheumatoid arthritis, Diabetes Mellitus), Low (Comorbidity burden). Stepwise regression included all listed sociodemographic, clinical, lifestyle, and disease predictors. Only variables that significantly improved model fit were retained. Model summary: R^2^=0.187, Adjusted R^2^=0.177, indicating that 17.7% of the variance in EQ-5D scores is explained by the predictors in the final model. Collinearity diagnostics confirmed low multicollinearity (all VIFs <1.5, condition indices <15).

Predictor Variable	B	SE	Standardized β	t-value	p-value	VIF
Constant	0.981	0.032	-	30.624	<0.001	-
Age (years)	-0.005	0.001	-0.310	-6.406	<0.001	1.4
Gender (Female vs. Male)	0.082	0.02	0.177	4.216	<0.001	1.06
Marital Status (Single/Divorced/Widowed vs. Married)	0.063	0.023	0.123	2.745	0.006	1.2
Rheumatoid Arthritis (Yes vs. No)	-0.177	0.043	-0.170	-4.101	<0.001	1.03
Diabetes Mellitus (Yes vs. No)	0.067	0.021	0.147	3.245	0.001	1.24
Comorbidity Burden (Moderate/High vs. Low)	-0.068	0.022	-0.149	-3.156	0.002	1.34

Age was negatively associated with EQ-5D index scores (B=-0.005, β=-0.310, p<0.001), indicating that HRQoL decreased with increasing age. Unlike in bivariate analysis, female gender was positively associated with EQ-5D index scores after adjustment for other variables (B=0.082, β=0.177, p<0.001). Regarding marital status, being single/divorced/widowed was associated with higher EQ-5D index scores compared to married participants (B=0.063, β=0.123, p=0.006).

Rheumatoid arthritis disease was significantly associated with lower EQ-5D index scores (B=-0.177, β=-0.170, p<0.001). Comorbidity burden was also associated with lower EQ-5D index scores (B=-0.068, β=-0.149, p=0.002). In contrast, diabetes was positively associated with EQ-5D index scores (B=0.067, β=0.147, p=0.001).

The final model explained 17.7% of the variance in EQ-5D index scores (adjusted R^2^=0.177). Collinearity diagnostics indicated no evidence of multicollinearity, as all VIF values were <1.5 and condition indices were <15.

## Discussion

The primary objective of this study was to evaluate the HRQoL and identify the associated factors among patients with chronic diseases in Saudi Arabia, using the EQ-5D-5L instrument. EQ-5D-5L is widely used to assess HRQoL due to its simplicity of converting the five-digit code of health status into one score that is useful for economic assessment and evaluation [4]. This is the first study in Jeddah conducted among chronic disease and multimorbidity patients using the EQ-5D-5L instrument to assess their HRQoL and compare it to a Saudi value set [10]. The selected chronic diseases to evaluate were the diseases with high prevalence among the Saudi citizens [1].

Overall HRQoL

This study found that patients with chronic diseases in Jeddah reported a moderate to good overall HRQoL, the overall mean EQ-5D index score was 0.84±0.23, and the mean EQ-VAS score was 77.22±20.60. Yet more than half of the participants reported imperfect health (301, 59.8%). Although the mean HRQoL scores suggest an overall acceptable health status at the population level, they may hide the large variability between individual patients' experiences. Specifically, a considerable proportion of patients continue to experience significant health limitations that are not fully reflected in average utility values. This finding is clinically important, as reliance on mean HRQoL scores alone may underestimate the true burden of suffering among patients with chronic diseases and might conceal the needs of those with the poorest health status. This supports recent findings of a similar multicenter Saudi study in the Al-Jouf region, which reported a consistent mean HRQoL of 0.82±0.20 for patients with chronic diseases [4]. This suggests a relatively stable national pattern of moderate HRQoL impairment in patients with chronic diseases across different geographical regions of Saudi Arabia.

The EQ-5D index and the EQ-VAS score showed a strong positive correlation (ρ=0.581). The consistency between these measures strengthens confidence in the HRQoL assessment and confirms the suitability of the EQ-5D-5L to evaluate health status among patients with chronic diseases in Saudi Arabia.

Dimension-specific HRQoL impairment

A detailed analysis of the EQ-5D-5L dimensions revealed that the primary drivers of this impaired HRQoL were pain/discomfort (mean score of 2.05±1.22) and anxiety/depression (mean score of 1.66±1.07). This is an important finding as the impact of chronic diseases in this population likely includes greater influence on symptom control and psychological well-being, rather than severe physical limitations. This pattern is not unique to this study, as it aligns strongly with both national and international literature, where chronic pain and psychological distress consistently emerge as dominant contributors to HRQoL decline in chronic disease populations [4,11].

The prominence of pain/discomfort is probably a reflection of the chronic deleterious effects associated with prevalent chronic diseases such as diabetes and cardiovascular disease. Persistent pain is a multifaceted issue that not only restricts physical functioning but also impacts daily living, sleep quality, and social engagement, thus exerting an additive effect on HRQoL [11]. This consistent pattern across regional and national studies highlights the necessity of integrated care models that prioritize specialized pain management and mental health support as essential components of chronic disease care in Saudi Arabia.

Similarly, elevated levels of anxiety and depression clearly highlight the significant psychological burden involved with living with a chronic disease. Fear of disease-related complications, uncertainty concerning long-term prognosis, and financial pressure all play a major role in this burden. Within the Saudi context, productivity pressures, family role responsibilities, and loss of personal independence have all been implicated in contributing to increasing levels of psychological distress reflecting mental health as a key but under-recognized aspect of chronic disease care [12,13].

The dominant role of multimorbidity

One of the most significant findings of this study is the strong negative association between multimorbidity and HRQoL. The HRQoL markedly declines in patients with multimorbidity. Participants with multiple chronic diseases reported a lower HRQoL compared to those with a single disease. Participants with a high disease burden (three or more chronic conditions) reported the lowest EQ-5D index score of 0.71±0.31 and EQ-VAS score of 63.6±21.2 (p<0.001). This finding aligns with growing evidence that multimorbidity represents a major challenge to both patients and healthcare systems, with prevalence rates in Saudi Arabia ranging widely depending on the population studied [1].

The decline in HRQoL among patients with multimorbidity may be due to the cumulative burden of managing multiple diseases. For each additional disease, there are commonly new symptoms, a complex and extensive treatment plan, and an increased frequency of complications, all leading to reduced functional independence of the patient [14]. This is further supported by a Saudi national study [10], which showed that individuals with multimorbidity reported more problems across all EQ-5D dimensions, especially in mobility and self-care.

Sociodemographic and lifestyle factors

Sociodemographic factors were found to be significantly associated with HRQoL in this study. Gender differences were evident. The mean EQ-5D index score was significantly higher in male participants than in female participants, who generally have lower HRQoL [15]. However, after adjusting for other variables, female gender was positively associated with higher EQ-5D index scores compared to male gender. This finding contrasts with previously conducted studies nationally and globally [16-19]. One possible explanation is that the initial bivariate differences were confounded by age and comorbidity burden. Studies in Saudi Arabia have shown that women are more likely to report multimorbidity compared to men, especially among older age groups [1].

Age also has an influence on HRQoL. In this study, the EQ-5D index score significantly decreases as the age increases. Younger participants (<30 years) reported the highest scores, while older participants (>60 years) reported the lowest. The negative correlation between age and HRQoL is widely reported across different geographical and clinical contexts. One study reported similar results in the general population of Germany with advanced age, which was associated with lower EQ-5D index score and EQ-VAS value [20]. This decline is often attributed to the physiological deterioration, reduced physical mobility, and greater burden of self-care [21]. Moreover, recent systematic reviews suggest that older individuals may interpret health states more negatively because of a substantial loss of independence [22].

Marital status showed significant associations with HRQoL, with single participants having higher EQ-5D index scores than married, divorced, or widowed participants. In the multivariable linear regression model, being single/divorced/widowed remained significantly associated with higher EQ-5D index scores compared to married participants, indicating an independent association after adjustment for other factors. This was also found by Alshammari et al. [16], and they attributed the higher EQ-5D index scores among single participants to the lower financial burden and fewer responsibilities they faced. One study reported conflicting results as they observed that married participants had the highest HRQoL compared to the other participants, and they linked this to the care and support they received from their family [23]. The higher EQ-5D scores among single participants may be attributed to their younger age, which is associated with fewer comorbidities and better overall health. Additionally, the family support within specific regions, such as in Saudi Arabia, may influence these outcomes, where single younger adults often remain within a supportive family before marriage [4].

The employment status of the participants has a positive impact on HRQoL, as employed participants reported the highest EQ-5D index score. However, students in this study reported the highest EQ-VAS score (90.04±15.11). Younger, student-aged populations often maintain high self-rated health despite the presence of chronic conditions. Students tend to rate their overall health status more favorably on a visual scale (EQ-VAS) than their specific functional limitations on the EQ-5D index [24]. Our findings regarding the benefits of employment differ from those reported in one study conducted in Riyadh among diabetic patients [23], which found that the unemployed participants have significantly higher HRQoL compared to the employed. This may resulted from the fact that employed participants might have faced higher levels of work-related stress, whereas the unemployed group may have had more time for self-care and disease management [23].

This positive relationship between income and HRQoL suggests that financial stability plays a crucial role in how patients perceive and manage their health. This is consistent with another study [10], which identified income as an important factor associated with health status. They observed that individuals with high income consistently reported better HRQoL compared to those with lower income. This is also in line with the results of a study conducted in the Hail region of Saudi Arabia [16], and they found that socioeconomic factors, including income, were significant predictors of HRQoL.

Furthermore, financial stability can reduce stress and anxiety related to managing the costs of long-term diseases, thereby having a positive impact on health. Similarly, lower income may limit access to necessary healthcare services, resulting in poorer perceived HRQoL [25,26]. Therefore, the results of this study emphasize that financial stability is not just a matter of financial status but is deeply related to the physical and mental health of patients with chronic diseases.

Clinical and disease-specific HRQoL

Disease type had an impact on HRQoL in this study. HRQoL scores were the lowest among patients with heart diseases and rheumatoid arthritis, whose lower HRQoL represents a loss of functioning and the considerable symptom burden associated with these conditions. Longer disease duration was also associated with worse HRQoL, which may be attributed to the chronic nature of illness with complications that exacerbate and accumulate over time, leading to treatment fatigue and diminished coping capacity [27].

Dedicated studies provide further insight into the influence of particular conditions. For example, among diabetic patients in Saudi Arabia, studies conducted using the EQ-5D-5L showed that those with complications (neuropathy, retinopathy) had significantly lower scores and that the presence of comorbid chronic diseases, such as hypertension, is associated with a further decline in health state [28]. This supports the concept that HRQoL is not entirely dependent on disease presence but is more closely related to disease severity, chronicity, and complexity of its complications.

Regarding diabetes mellitus, the positive association observed in the adjusted model should be interpreted within the context of the study population, which consisted exclusively of patients with chronic diseases. Thus, the comparison in this study was between individuals with diabetes and those with other chronic conditions, rather than healthy individuals. It is possible that some of the other chronic conditions included in this study, especially those characterized by significant pain and functional limitation, may have a more negative impact on HRQoL than diabetes, especially in the case of well-managed diabetes and in the absence of severe complications.

Disease duration and HRQoL

Mean disease duration was associated with low EQ-5D index score and EQ-VAS score. However, the results were not statistically significant. This finding indicates that the length of time patients have lived with their chronic condition may contribute to reducing their quality of life, although not as strongly as other factors, such as multimorbidity and socioeconomic status, in affecting HRQoL. Our results are consistent with previous studies; for instance, one study conducted in Riyadh [29] found no significant association between disease duration and HRQoL scores among diabetic patients. Likewise, a study from the Eastern region [19] found no significant differences in HRQoL with respect to the duration of disease in diabetic patients. The lack of statistical significance in these findings may be explained by the observation that over time, many patients with chronic diseases learn to adapt to their condition and adjust their health expectations [30].

The findings from this study provide evidence to support the need for national strategies that prioritize quality of life outcomes, not only disease control indicators. Healthcare providers should focus particularly on chronic disease patients with multiple comorbidities, as the number of comorbidities is the strongest predictor of poor HRQoL. Interventions should be specifically targeted at older patients, females, and those with low socioeconomic status, as these groups were identified as having significantly lower HRQoL.

From a health policy and economic perspective, the national adoption of the Saudi-specific EQ-5D-5L value set is strongly recommended for health technology assessment and pharmacoeconomic evaluations. Utilizing locally derived utility values will enhance the accuracy of cost-effectiveness analyses, support evidence-based resource allocation, and ensure that funding decisions reflect the true health preferences and quality of life priorities of the Saudi population.

One of the main strengths of this study is its large sample size and multicenter design. In addition, the use of a stratified geographic sampling approach further strengthens the methodology by ensuring representation from different regions of Jeddah and reducing the risk of selection bias. The use of a validated and standardized HRQoL instrument (EQ-5D-5L) and the application of the Saudi value set further strengthen the methodological quality. Additionally, the inclusion of a wide range of chronic diseases and comprehensive variables provides a broad assessment of HRQoL.

This study has several limitations that are important to acknowledge. First, as a result of budget and time constraints, a cross-sectional design was employed in the study, which may prevent causal inference between chronic diseases and HRQoL. Second, the study was conducted only on patients in Jeddah. Although the sample is quite representative, results may not be fully generalizable to the entire Saudi population, especially those from rural and remote areas who have different healthcare accessibilities and socioeconomic features. In addition, the use of self-reported data introduces the possibility of recall bias, since participants may fail to remember or report certain aspects of disease duration, lifestyle, and clinical characteristics correctly. This may have affected the accuracy of some associations in the study. Income data were missing for 177 participants (35.2% of the total sample). Therefore, an analysis of income and its association with HRQoL was conducted for 326 participants. This high proportion of missing income data may introduce potential bias and should be considered when interpreting these findings. Finally, other potential predictors like severity of disease, adherence to treatment, and mental health diagnosis were not investigated.

## Conclusions

This study revealed that patients with chronic diseases in Jeddah generally reported moderate to good HRQoL, the overall mean EQ-5D index score was 0.84±0.23, and the mean EQ-VAS score was 77.22±20.60. More than half of the participants experienced impaired health, especially in pain and psychological well-being. HRQoL is largely affected by sociodemographic, clinical, lifestyle, and disease-related characteristics. Multimorbidity was the most significant cause of impaired HRQoL, whereas the disease duration was not significantly related to HRQoL.

While this study provides important local evidence using a culturally specific value set, the cross-sectional design limits the causal inference. Therefore, associations observed between multimorbidity and HRQoL should not be interpreted as causal relationships. Longitudinal studies are recommended to further explore temporal relationships and the long-term impact of disease burden on HRQoL. These findings underscore the need for a patient-centered, integrated strategy to manage chronic diseases and provide important evidence that can help guide clinical practice, health policy, health economic decisions, and future research in Saudi Arabia.
